# Supplementary material for: Causal Relationship between Aging and Anorexia Nervosa: A White-Matter-Microstructure-Mediated Mendelian Randomization Analysis
Source: Biomedicines. 2024 Aug 16;12(8):1874. doi: 10.3390/biomedicines12081874 (PMC11351342; doi:10.3390/biomedicines12081874)
Supplement: Supplementary file 1 [file biomedicines-12-01874-s001.zip › Supplementary Figure/Figure S9/Leaveoneout plot of MR analysis for ubm-b-1852 to AN.pdf]

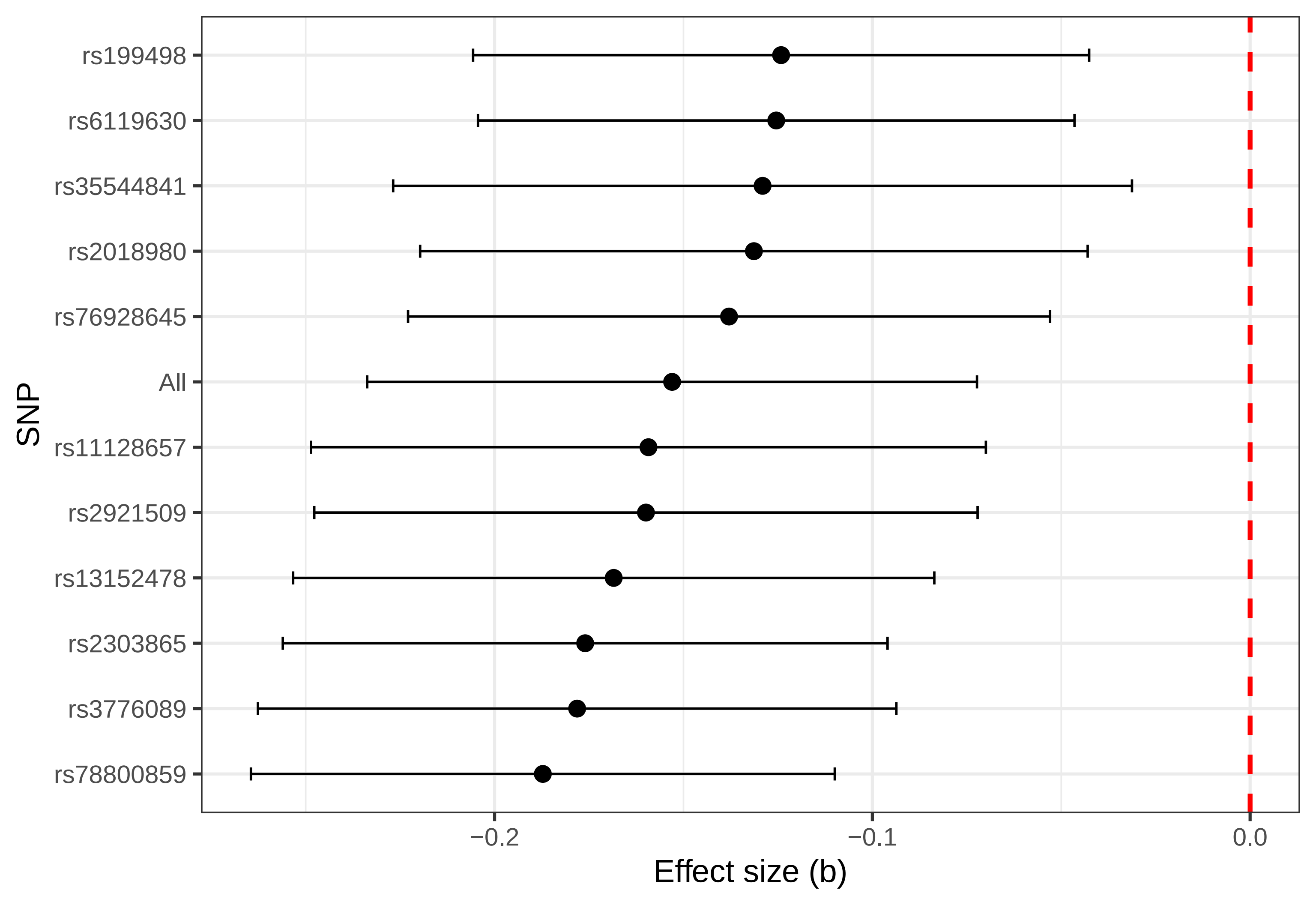

rs199498
rs6119630
rs35544841
rs2018980
rs76928645
All
rs11128657
rs2921509
rs13152478
rs2303865
rs3776089
rs78800859
SNP
−0.2
−0.1
0.0
Effect size (b)
